# Silent Paralysis: Recurrent Thyrotoxic Periodic Paralysis in a Young Hispanic Male With Graves’ Disease

**DOI:** 10.1016/j.aed.2025.02.005

**Published:** 2025-04-10

**Authors:** Rouba Isshak, Nagihan Orhun, Islam Rajab, Dale Shober, Patrick Michael

**Affiliations:** Department of Internal Medicine, St. Joseph’s University Medical Center, Paterson, New Jersey

**Keywords:** thyrotoxic periodic paralysis (TPP), Graves’ disease, euthyroid state, rebound hyperkalemia, nonselective β-blockers

## Abstract

**Background/Objective:**

Thyrotoxic periodic paralysis is a rare, life-threatening complication of thyrotoxicosis, characterized by hypokalemia, hyperthyroidism, and acute muscle weakness. It often occurs in Graves’ disease but is not directly linked to the severity or duration of thyrotoxicosis. The objective of this report is to describe a patient with Graves’ disease and thyrotoxic periodic paralysis, emphasizing the importance of achieving an euthyroid state, potassium repletion, and the role of β-blockers in management.

**Case Report:**

A 23-year-old male with a history of untreated hyperthyroidism presented to the emergency department with palpitations and worsening bilateral lower extremity weakness, culminating in an inability to walk.

The patient reported previous episodes of weakness associated with his hyperthyroidism. Physical examination revealed marked muscle weakness and hypertension. Following evaluation, the patient was diagnosed with Graves’ disease. He was treated with potassium, magnesium, methimazole, and propranolol, resulting in rapid improvement of his symptoms, and was discharged with outpatient follow-up.

**Discussion:**

Thyrotoxic periodic paralysis is a rare manifestation of thyrotoxicosis, which can be precipitated by stressors or medication noncompliance. The pathophysiology involves thyroid hormone-induced shifts in potassium, leading to hypokalemia and muscle weakness. Treatment consists of normalizing thyroid function, managing potassium, and using β-blockers to counteract the overstimulation of muscle cells by thyroid hormones and catecholamines. However, the precise role of β-blockers in preventing attacks requires further investigation.

**Conclusion:**

Early diagnosis and treatment of thyrotoxic periodic paralysis, including euthyroid restoration and potassium management, are critical. While β-blockers may be effective in symptom resolution, further studies are needed to clarify their role in preventing attacks.

**Highlights**

• Thyrotoxic periodic paralysis (TPP) is a rare but potentially life-threatening condition
• Achieving an euthyroid state is critical in the management of TPP
• Potassium repletion is essential, but overcorrection can be dangerous
• Nonselective β-blockers may help in potassium-resistant cases of TPP
• Further research is needed to understand the exact role of β-blockers

**Clinical Relevance**

Early diagnosis and treatment of thyrotoxic periodic paralysis are essential to prevent life-threatening complications, particularly arrhythmias. Further investigation is required to understand the role of β-blockers in preventing or aborting attacks.

## Introduction

Thyrotoxic periodic paralysis (TPP) is a rare but severe complication in patients with thyrotoxicosis. It is marked by hypokalemia, which causes acute proximal symmetrical weakness. TPP typically affects the lower limbs and may progress to involve all 4 limbs and respiratory muscles, and is considered exceedingly rare.^1^

Paralysis can arise from any cause of hyperthyroidism, including Graves’ disease, toxic nodular goiter, solitary toxic nodule, iodine-induced thyrotoxicosis, or excessive use of exogenous thyroxine.^2^

In this report, we present a case of TPP in a patient who was diagnosed with Graves’ disease.

## Case Report

A 23-year-old male with a history of hyperthyroidism presented to the emergency department with complaints of palpitations and generalized weakness affecting the bilateral lower extremities. The patient was diagnosed with hyperthyroidism 2 years before the presentation, but had not been taking any medication. Upon evaluation, the patient reported a 2-day history of progressive tingling sensations, muscle cramps, and lower extremity weakness, which became markedly worse within hours before coming to the hospital, leading to an inability to walk, which prompted him to call the Advanced Life Support team. A review of the systems was otherwise unremarkable. The patient reported a similar episode of severe lower extremity weakness, in which he was hospitalized and diagnosed with hyperthyroidism. He denied any recent infections, travel, or sick contacts.

Initial vitals were significant for hypertension (blood pressure: 158/96 mm Hg) and tachycardia (heart rate: 110 bpm). The patient was alert and oriented to person, place, and time on the physical examination. Strength was 1/5 in bilateral lower extremities and 3/5 in bilateral upper extremities, with decreased muscle tone in bilateral lower extremities compared to upper extremities; deep tendon reflexes were diminished; and sensation to light touch and pain was intact in all extremities. Cranial nerves II-XII were intact. The electrocardiogram showed sinus tachycardia with flattening of the P wave and prolonged QTc of 504 ms. Labs showed severe hypokalemia (K:1.9 mEq/L), hypomagnesemia (Mg:1.6 mg/dL), and hypophosphatemia (1.4 mg/dL). Thyroid-stimulating hormone, free T4, and free T3 returned <0.01 mcIntlUnit/mL, 2.88 ng/dL, and 7.68 pg/mL, respectively. Burch-Wartofsky score was 15, indicating that a thyroid storm was unlikely. CT head without contrast showed no acute intracranial abnormality.

Aggressive potassium and magnesium repletion was initiated, with a complete resolution of the patient’s muscle weakness over 12 hours. The patient also started on methimazole 40 mg daily, cholestyramine 4 g three times a day, and propranolol 60 mg every 6 hours with a plan to titrate based on heart rate. Thyroid ultrasound was done, and it showed an abnormally enlarged thyroid with increased vascularity. Thyroid-stimulating immunoglobulin and anti-thyroglobulin antibodies returned elevated 0.79 (0-0.55 IU/mL) and 210 (0-0.9 IU/mL), respectively. The patient was then diagnosed with Graves’ disease, which induced TPP due to medication noncompliance for a few months before presentation, and was discharged on methimazole 40 mg daily for 4 weeks with a plan to taper down to 20 mg daily afterward with outpatient endocrinology follow-up.

## Discussion

TPP is a rare yet potentially life-threatening consequence of thyrotoxicosis. It is a clinical and biochemical diagnosis in which patients demonstrate a triad of hypokalemia, hyperthyroidism, and acute onset of muscle weakness.^2^ While it frequently occurs in Graves’ disease, the occurrence of TPP is not directly linked to the cause, severity, or duration of thyrotoxicosis.^2^ According to a Chinese study, 2% of patients with thyrotoxicosis in China and Japan are reported to have TPP.^3^ However, in the United States, the incidence of TPP among the non-Asian population is around 0.1% to 0.2%. This difference is attributed to genetic admixture, population mobility, and insulin resistance, which are attributed to the rising obesity epidemic.^4^

Additionally, genetic mutations can increase susceptibility to TPP, with the most noted mutation affecting the gene encoding Kir2.6, an inwardly rectifying potassium channel regulated by thyroid hormone.^5^

Presentations of TPP can vary; approximately half of patients may not exhibit obvious symptoms of hyperthyroidism during an episode,^2^ with some individuals showing subtle or atypical signs of thyroid dysfunction.^6^

Clinically, patients report a history of recurrent muscle aches, cramps, and stiffness. Weakness usually starts in the proximal muscles of the lower extremities and can progress to flaccid quadriplegia with hypotonia and hyporeflexia.^2^ However, in the literature, a TPP case presented with increased tone and hyperreflexia improved after the episode was resolved.^7^ In our case, it was the opposite, and the patient presented with the typical picture of TTP. Paralysis is usually symmetrical but can be asymmetrical or limited to overexerted muscles. Mental function, sensation, bulbar, respiratory, and ocular muscles are typically unaffected.^2^

TPP attacks often occur at night or early in the morning, with increased frequency during the summer months.^2^^,^^8^ During these episodes, symptoms can last from several hours to days.^9^ These attacks are linked to significant shifts of extracellular potassium into muscle tissue and are often precipitated by a stressor on the body; potential triggers include strenuous exercise, high-carbohydrate meals, trauma, infections or acute illnesses, trauma, cold exposure, menstruation, and emotional stress.^2^ Notably, nearly 34% of cases have no identifiable precipitating factors.^10^ In our case, the primary precipitating factor was medication noncompliance for at least 4 months before presentation.

In addition to muscle paralysis, severe electrolyte imbalances in TPP, particularly potassium deficit, can cause life-threatening cardiac arrhythmias and cardiomyopathy by disrupting normal cardiac conduction.^11^

Thyroid hormones profoundly enhance β-adrenergic receptor sensitivity in skeletal muscle, increasing responsiveness to catecholamines like epinephrine, released during stressful situations or triggers. When epinephrine binds to β2-adrenergic receptors on skeletal muscle cells, it activates adenylyl cyclase, increasing cAMP levels and activating protein kinase A and phosphorylation of ion channels, including voltage-gated calcium and potassium channels.^5^ This cascade results in a massive potassium efflux from the muscle cells, contributing to hypokalemia, affecting muscle function, and leading to the characteristic paralysis in TPP.^12^

Diagnosis involves identifying hypokalemia with an average acid-base balance alongside suppressed thyroid-stimulating hormone with elevated T3 and T4 levels. Potassium levels correlate with the severity of muscle weakness; however, no correlation exists between T3 and T4 levels.^13^

The significance of our discussion is to focus on the optimal treatment for TPP, as the first crucial step is achieving an euthyroid state. For patients with Graves’ disease, the recommended initial treatment involves anti-thyroid medications followed by either radioactive iodine therapy or thyroidectomy.^14^ In addition, acute potassium replacement is vital for normalizing serum potassium levels to prevent life-threatening arrhythmias. Nevertheless, care must be taken to avoid overcorrection, and patients should be closely monitored for rebound hyperkalemia, which occurs in about 40% of cases and can be fatal.^2^^,^^15^ The case study by Polamaung et al^16^ noted that potassium administration in the first 24 hours should not exceed 90 mEq due to the risk of rebound hyperkalemia.^17^ Additionally, correcting magnesium is essential in cases of hypokalemia, as hypomagnesemia increases the risk of dangerous ventricular arrhythmias.^16^

Some patients may not respond adequately to potassium treatment; in such instances, intravenous propranolol has been effective.^18^^,^^19^ β-blockers, specifically propranolol, are commonly used to treat TPP by blocking the β2-adrenergic receptors, interrupting the whole cascade of TPP pathophysiology. This reduces cAMP production, decreases protein kinase A activation, and stabilizes the potassium gradient, reducing potassium efflux and preventing hypokalemia.^18^^,^^19^ Propranolol blocks both β1 and β2 receptors, effectively preventing catecholamine-induced potassium shifts in skeletal muscle, which are central to TPP pathophysiology.^18^^,^^19^ Moreover, by blocking β1 receptors, propranolol can reduce the sympathetic nervous system response, helping stabilize the cardiac function in TPP patients at risk for arrhythmias.^19^ Selective β1 blockers, like atenolol or metoprolol, are used for cardiac conditions and are less effective in managing TPP. Moreover, carvedilol, despite blocking β2 receptors, is not routinely used due to its α1-blocking effects on blood pressure, although it may be considered for patients with hypertension.

Studies present mixed views on the role of nonselective β-blockers. Some suggest they terminate paralysis once attacks begin. In contrast, others argue that they may be a preventive measure against paralytic episodes.^17^ Notably, case reports indicate that some patients on propranolol were still susceptible to paralysis. Therefore, further investigation is needed to clarify the precise role of β-blockers in preventing TPP attacks.

## Conclusion

In conclusion, TPP is a rare, potentially fatal complication of thyrotoxicosis, typically affecting the lower limbs, which may also affect the upper limbs and muscles of respiration. Careful history, examination, and understanding of TPP’s key epidemiologic features are crucial for early diagnosis. During treatment, it is essential to avoid the common and potentially dangerous consequences of potassium overcorrection and allow the patient to receive propranolol and definitive anti-thyroid treatment with minimal delay. Nonselective β-blockers may be used for hypokalemia resistant to potassium treatment or as an adjacent therapy, as was done in this case. However, the role of these medications is unclear in the literature. Further research is needed to clarify their treatment of TPP attacks as preventative or abortive.

## Disclosure

The authors have no conflicts of interest to disclose.
